# A process for purifying xylosugars of pre-hydrolysis liquor from kraft-based dissolving pulp production process

**DOI:** 10.1186/s13068-018-1336-0

**Published:** 2018-12-21

**Authors:** Jiachuan Chen, Jiran Dong, Guihua Yang, Ming He, Feng Xu, Pedram Fatehi

**Affiliations:** 1 State Key Laboratory of Bio-based Materials and Green Papermaking, Qilu University of Technology (Shandong Academy of Sciences), Jinan, 250353 Shandong China; 2 Green Processes Research Centre and Department of Chemical Engineering, Lakehead University, Thunder Bay, ON P7B 5E1 Canada

**Keywords:** Pre-hydrolysis liquor, Ca(OH)_2_, Lignin removal, Activated carbon, Laccase, Xylosugars

## Abstract

**Background:**

In the kraft-based dissolving pulp production process, pre-hydrolysis liquor (PHL) is produced, which contains hemicelluloses, lignin, furfural and acetic acid. PHL is currently burned in the recovery boiler of the kraft pulping process, but it can be utilized for the generation of high-valued products, such as xylitol and xylanase, via fermentation processes. However, some PHL constituents, e.g., furfural and lignin, are contaminants for fermentation processes and they must be eliminated for production of value-added products.

**Results:**

In this work, a process is introduced for removing contaminants of PHL. Ca(OH)_2_ treatment is the first step of this process, which removed 41.2% of lignin and negligible amount of sugars. In this step, a notable increase in the concentration of acetic acid was achieved (ranging from 6.2 to 11.7 g/L). In the second step, the implementation of adsorption using activated carbon (AC) at 1 wt% dosage led to additional 32% lignin and 5.9% xylosugar removals. In addition, laccase assisted activated carbon treatment led to further removal of lignin via accelerating lignin polymerization and adsorption on AC (i.e., removal from PHL). Overall, 90.7% of lignin, 100% of furfural, 5.7% of xylose, and 12% of xylan were removed from PHL, while the concentration of acetic acid became twofolds in the PHL.

**Conclusions:**

This study reports an attractive process for purifying sugars and acetic acid of PHL. This process may be implemented for producing sugar-based value-added products from PHL. It also discusses the mechanism of Ca(OH)_2_ treatment, AC adsorption and laccase assisted activated carbon treatment for lignin removal.

## Background

In the hydrolysis stage of kraft-based dissolving pulp process, most of the hemicellulose and part of lignin are dissolved into liquor that is called pre-hydrolysis liquor (PHL). Currently, PHL is considered a waste liquor and mixed with black liquor for combustion in the recovery boiler of the kraft process [1, 2]. However, because of its dilute nature and its low heating value, its combustion is unprofitable [3].

Previous studies showed that the PHL from the kraft-based dissolving pulp production process generally contains 3–5% organics, which can be converted to value-added products, such as ethanol and xylitol [4]. Moreover, hemicellulose, e.g., xylose, is widely used in the food and medicine industries owing to its ulcer protection [5], antitussive [6] and immunostimulatory [7] effects. However, the presence of lignin in the PHL hinders the utilization of hemicelluloses. For example, lignin hinders sugar fermentation to produce ethanol or xylitol [8]. Therefore, finding an effective approach to eliminate contaminants is crucial for generating hemicellulose-based value-added products from PHL.

In the past, solvent extraction was employed to separate/isolate hemicellulose from PHL [9]. Despite its high efficiency, the complexity of solvent recovery, the high operational costs and technical issues constrained the commercialization of this technology [1]. Acidification has also been proposed to isolate lignin from PHL, but its effectiveness was defective [10]. Other methods were also studied for purifying pentoses from PHL including (i) physical treatments, such as membrane extraction and activated carbon (AC) treatment [1, 11]; (ii) chemical treatments, such as poly dimethyl diallyl ammonium chloride (PDADMAC) treatment; and (iii) biochemical treatments, such as laccase treatment [12]. Membrane extraction is effective, but it has a major filter blockage and is costly to operate [13]. PDADMAC is poor in removing lignin, it is an expensive chemical and it may cause environmental issues [14]. Therefore, a more effective method should be developed for removing/isolating contaminants from PHL for facilitating hemicellulose usage.

In one study, lime treatment was an effective approach for removing lignin from PHL, but an extensive sugar degradation was reported at a high dosage of lime in PHL [15]. Lime treatment may be an acceptable option if it is used at a low dosage, as it is well in harmony and is actually used in the kraft-based pulping process. Therefore, the first objective of this work is to study whether Ca(OH)_2_ treatment can benefit the utilization of hemicelluloses of PHL.

Activated carbon (AC) has been vastly used as an adsorbent in industry [16]. It is a widely available and effective material for adsorption of lignin [17]. However, AC adsorbs both lignin and hemicelluloses [1]. In the past, AC was used to adsorb lignin and sugar from a mixed solution [3]. Lignin is hydrophobic, but sugars are hydrophilic, therefore, the adsorption of these chemicals on AC may be poor. The second objective of this work was to investigate if AC addition can be combined with other methods to increase its selectivity towards adsorbing PHL contaminants.

Recently, laccase, a copper-containing oxidase, has received attention for its sustainable selectivity for lignin removal under mild reaction conditions. It is known that laccase treatment can increase lignin’s molecular weight [18]. This is attributed to the fact that laccase can polymerize small lignin molecules to make them more hydrophobic, which can enhance lignin removal from PHL [1, 12]. The third objective of this work is to investigate the effect of laccase treatment on lignin removal from PHL.

In the past, some process operations were suggested for removing lignin from PHL, but these processes had limited removal efficiencies [1, 15, 18]. The main novelty of this work is the design of an attractive process via combining single state processes for efficiently separating contaminants from PHL, while maintaining xylosugars and acetic acid in the PHL for their possible utilization in downstream biochemical or chemical processes.

## Methods

### Materials

Industrially produced pre-hydrolysis liquor (PHL) was collected from a kraft-based dissolving pulp mill located in eastern China. Eucalyptus wood chips were used as raw materials. The pre-hydrolysis was performed at 170 °C for 60 min as it practiced at the mill. The chemical constituents of PHL are listed in Table 1. Ca(OH)_2_ (particle size 5 μm), H_2_SO_4_, dioxane, ethyl ether, pyridine, acetic anhydride, tetrahydrofuran (THF) and P_2_O_5_ were all analytical grades and purchased from Tianjin Hengxing Company. Activated carbon was obtained from Guangzhou Haiyan Company. Laccase and 2, 2′-azino-bis (3-ethylbenzthiazoline-6-sulfonate) (ABTS) were purchased from Sigma-Aldrich. Laccase activity was determined by using a UV–vis spectrophotometer (Agilent Technologies, Palo Alto, America) at 420 nm and pH 4.5 at 20 °C with 0.5 mM ABTS as the substrate, as described by Mansfield [19]. One activity unit (U/mL) was defined as the amount of enzyme that oxidized 1 μmol of ABTS in a minute.

**Table 1:** Chemical constituents of original PHL

Monomeric form (g/L)		Oligomeric form (g/L)		Other products (g/L)	
Xylose	12.3 ± 0.68	Xylose	23.3 ± 0.79	Soluble lignin	9.7 ± 0.49
Arabinose	0.2 ± 0.005	Arabinose	0.1 ± 0.006	Acetic acid	6.2 ± 0.26
Galactose	0.4 ± 0.013	Galactose	1.4 ± 0.067	Furfural	1.9 ± 0.1
Glucose	0.4 ± 0.017	Glucose	1.2 ± 0.055	HMF	0.4 ± 0.032
Mannose	0.3 ± 0.006	Mannose	1.0 ± 0.03		
Total	13.6 ± 0.721	Total	27.0 ± 0.948	Total	18.2 ± 0.453

*HMF* hydroxymethylfurfural

**Fig. 1:**
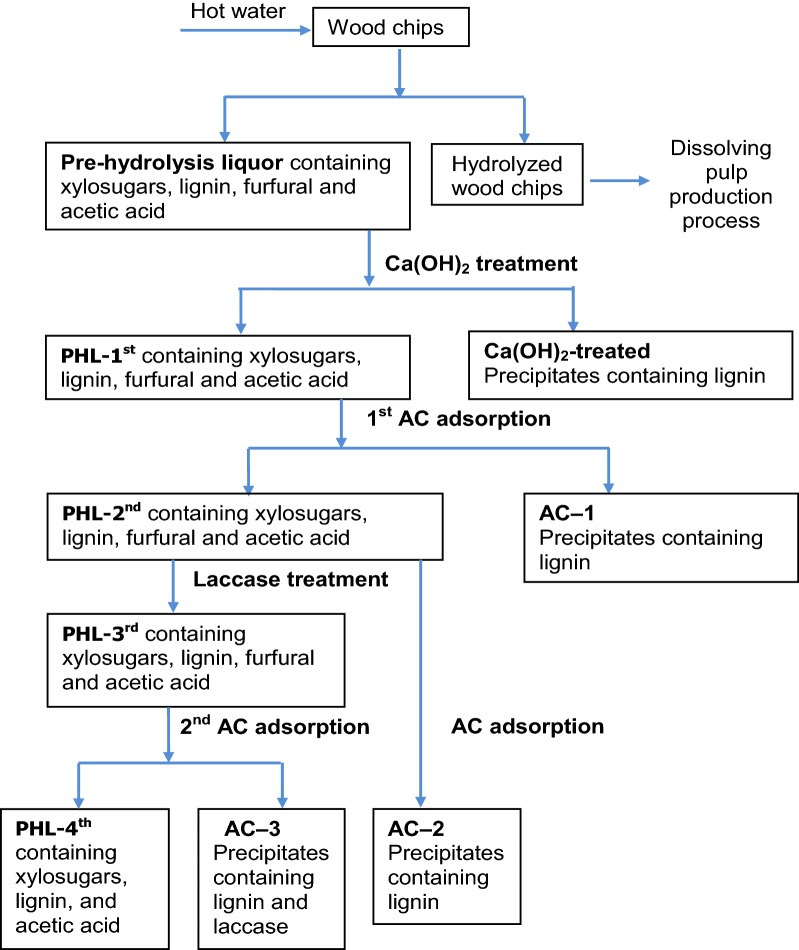
Experimental procedure for removing contaminants and purifying xylosugars

Figure 1 shows the flow chart of the experimental procedure to purify xylosugars of the PHL. As seen in Fig. 1, PHL was collected from hot water hydrolysis process and the hydrolyzed wood chips were subsequently used to produce dissolving pulp. PHL was treated by Ca(OH)_2_, AC and laccase assisted AC for removing contaminants from PHL (i.e., purifying xylosugars in PHL).

### Ca(OH)_2_ treatment of PHL

Ca(OH)_2_ treatment experiments was conducted at 25 °C. Different amounts (0.5–4.0 g) of Ca(OH)_2_ were added into glass flasks containing 100 g of PHL. The mixtures were stirred at 250 rpm for 5 min at room temperature. These conditions were selected according to literature results and process optimization (not shown) [15]. The treated liquors were centrifuged at 4500 rpm for 3 min. The precipitates (containing calcium hydroxide particles) were collected for Fourier transform infrared spectroscopy (FT-IR) analysis. The first filtrates of PHL were neutralized to pH 7 by using H_2_SO_4_ for forming precipitates of CaSO_4_ and then were centrifuged to obtain the filtrates of PHL, which were denoted as PHL-1st (Fig. 1). Each experiment was repeated three times to ensure the experimental repeatability. The error bars show the variations in the experimental results.

### 1st AC adsorption stage

The adsorption experiments were performed at 25 °C with adding different dosages of activated carbon (0.5 wt%, 1.0 wt%, 2.0 wt%, 4.0 wt% and 6.0 wt%) to 100 g of PHL-1st in glass flasks. The agitation was performed at 150 rpm for 5 min (Fig. 1). Then, the samples were filtered using regular filter papers. The filtrates of this process were collected as PHL-2nd. The precipitates were collected as AC-1. It should be stated that the adsorption of PHL components on the filter papers used in this study was tested, and the results confirmed negligible removals. Therefore, filtration had minimal impact on the removal of PHL components.

## Laccase assisted AC treatment

### Laccase treatment

Laccase treatment experiments were carried out in Erlenmeyer flasks at 150 rpm of shaking rate and different treating conditions. The dosages of laccase varied from 0 to 10 U/g in 30 g of PHL-2nd. The pH of this treatment ranged from 4 to 8. The treating temperature and time were 25–55 °C and 1–5 h, respectively. In this test, the mixture was boiled for 10 min to deactivate laccase after laccase treatment. The mixtures were collected as PHL-3rd. The effect of laccase treatment on the average molecular weight (Mw) and number-average molecular weight (Mn) of lignin of the PHL before (for PHL-2nd) and after (for PHL-3rd) this treatment was evaluated.

### 2nd AC adsorption stage

The PHL-3rd obtained under the optimal laccase treatment conditions was mixed with 0.2 wt% to 1.2 wt% of AC. The other conditions of this AC adsorption stage were same as those of the first AC adsorption stage. After the treatment, the mixtures were filtered using filter papers and the collected filtrate was denoted as PHL-4th. The collected treated AC was denoted as AC-3 and its surface area and pore structure were analyzed.

To understand the influence of laccase treatment on the contaminant removal, 1 wt% of AC was added into PHL-2nd, and the treated AC of this step was denoted as AC-2 after collection. The other experimental conditions in this step were the same as those followed for producing AC-3, but without the laccase treatment.

### Chemical component analysis

The monosaccharide concentrations in PHL were determined by an ion chromatography (ICS-5000+, Thermo Scientific, America), which was equipped with a Dionex CarboPac PA20 column (3 mm × 150 mm, Thermo Scientific, America) and an ED40 electrochemical detector. Samples were run at 30 °C and the flow rate was 0.4 mL/min in this instrument. The mobile phase used in the chromatography consisted of 4% of 50 mmol/L NaOH and 96% of ultrapure water for a period of 22 min. Then, the eluent was switched to 40% of 1 mol/L NaCOOCH_3_, 20% of ultrapure water and 40% of 50 mmol/L NaOH for another 5 min; and it was finally switched to 20% of ultrapure water and 80% of 250 mmol/L NaOH for another 28–35 min. For measuring the concentration of oligosaccharides in solutions, an additional acid hydrolysis step was carried out on the collected samples under the conditions of 4% sulfuric acid concentrations at 121 °C for 1 h in an oil bath. Then, the concentrations of sugars in the acidified samples were determined following the method stated above. The concentration of oligosaccharides was determined via subtracting the concentration of monosaccharides before this acid hydrolysis step from that obtained after the acid hydrolysis stage.

The concentrations of acetic acid, furfural and hydroxymethylfurfural (HMF) in the solutions were measured by a Shimadzu LC-20T high performance liquid chromatography (HPLC) system (Japan), which was equipped with a SUPELCOGEL C-610H column (30 cm × 7.8 mm, Sigma-Aldrich, America) and a SPD-20A detector. Samples were run at 30 °C and eluted at 0.7 mL/min, and mobile phase was 0.1% H_3_PO_4_. The samples were filtered using a 0.22 μm Nylon syringe filter prior to the analysis.

The concentration of lignin in solutions was determined by a UV/vis spectrophotometer (Agilent Technologies, Palo Alto, America) according to the TAPPI standard TAPPI UM 250 method at wavelength of 205 nm [20].

### Particle size analyses

The particle size of the components in PHL was measured by a particle size analyzer, Malvern Zetasizer Nano ZSP (Malvern, UK). The scattering angle and operating wavelength were 90° and 658 nm, respectively. The mean diffusion coefficient was obtained using a dynamic light scattering (DLS) (Malvern, UK) measurement. The hydrodynamic sizes of the components in PHL were calculated following Stokes–Einstein Equation [21]. The sample was diluted 50 times and filtered using 0.22 µm nylon filters before the measurement.

### Lignin isolation

The lignin of the PHL untreated and treated by laccase was isolated by following the method described in the literature [22]. The PHL was acidified to pH 2 by H_2_SO_4_, and its lignin was precipitated and separated by centrifugation at 5000 rpm. The precipitated lignin sample was thoroughly washed with distilled water to neutrality. Then, the lignin obtained after washing was dissolved in dioxane (9/1 vol/vol) and precipitated in ether under constant stirring. The precipitated lignin was dried in vacuum over P_2_O_5_.

### Acetylation and gel permeation chromatography

The lignin samples obtained previously (200 mg) were acetyl-brominated in a mixture of 10 mL of dry pyridine/acetic anhydride (1/2 vol/vol) and kept in the dark for 72 h (room temperature, 25 °C). Once the bromination was complete, the solutions were added to a tenfold volume of ethyl ether. The mixture was centrifuged, and the acetylated lignin was recovered as precipitates. Subsequently, the precipitates were purified by successive washing with ethyl ether and dried under vacuum over P_2_O_5_. The purified lignin was dissolved in tetrahydrofuran (THF). Then, the weight average (Mw) and number average molecular (Mn) weights of acetylated lignin were determined by a gel permeation chromatography (GPC) using a Shodex KF-802.5 column with UV detection (280 nm) and differential refractive index (RI) detection. The analyses were carried out at 30 °C using THF as the eluent at the flow rate of 1 mL/min.

### Characterization of Ca(OH)_2_ and activated carbon

The surface functional groups of Ca(OH)_2_ and AC before and after treatment were characterized by using a Fourier transform infrared spectrometer (VERTEX70, Bruker, Germany). The oven-dried samples were embedded in KBr pellets in a mixture of about 1 wt% KBr. The spectra were monitored in a transmittance band mode in the range 500–2500 cm^−1^.

The BET surface area and pore structure characteristics of original AC, AC-2 and AC-3 were determined by using a surface area analyzer (v-sorb2800p, Gold APP Instrument Corporation, Beijing, China) via nitrogen adsorption/desorption isotherms. About 0.1 g of AC was pretreated at 110 °C overnight for contamination removal. Afterward, the measurement was carried out using nitrogen as a probe at 77 K overnight.

### High heating values (HHV) analysis

Organic elements (C, H, O, N and S) of AC before and after treatment were estimated by using a Vario EL III Elementar (USA). About 2.5 mg of sample was pretreated at 105 °C overnight for moisture removal. High heating value (HHV) was calculated following Eq. 1 [23].

1 $${\text{HHV}}\left( {\text{MJ/kg}} \right) = 0. 3 4 9 1 {\text{C}}\, + \, 1. 1 7 8 3 {\text{H}}\, + \,0. 100 5 {\text{S}} - 0. 10 3 4 {\text{O}} - 0.0 1 5 1 {\text{N}} - 0.0 2 1 {\text{A}}$$

where, C, H, S, O, N, and A represent carbon, hydrogen, sulfur, oxygen, nitrogen, and ash content (wt%) of the samples, respectively.

## Results and discussion

### Chemical analysis of original PHL

The chemical constituents of the original PHL are shown in Table 1. It can be seen that xylosugars were the primary components of PHL as hardwood was used in the dissolving pulp production process, and these findings are similar to those reported earlier [3, 24].

### Ca(OH)_2_ treatment

**Fig. 2:**
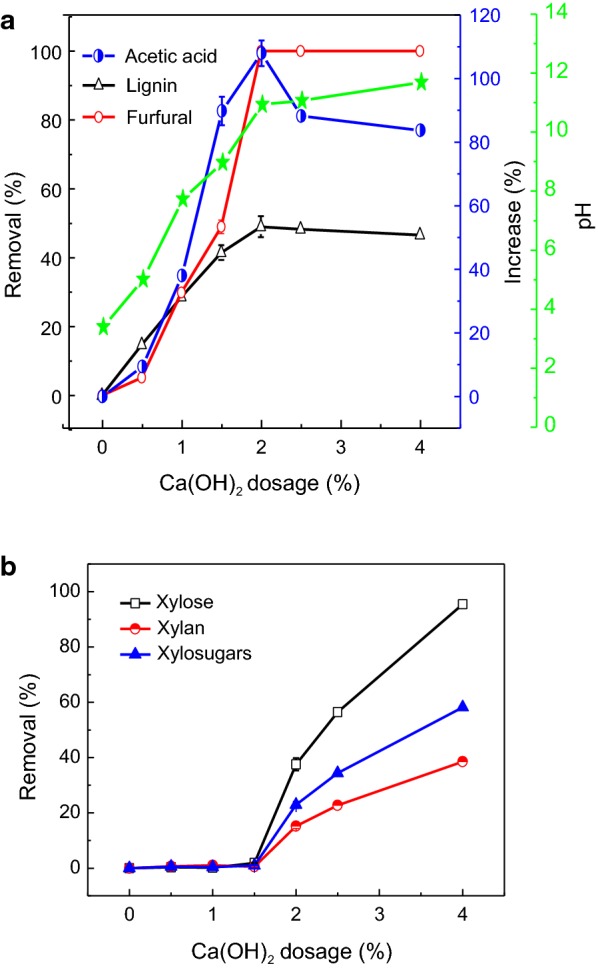
Effect of Ca(OH)_2_ dosage on the constituents of original PHL (**a** removal of lignin and furfural, and increase of acetic acid; **b** removal of xylose, xylan and xylosugars)

The effect of Ca(OH)_2_ dosage on the removal of acetic acid, lignin and furfural in PHL is shown in Fig. 2a. The pH of the PHL increased rapidly with increasing Ca(OH)_2_ dosage. However, the pH was changed negligibly when Ca(OH)_2_ dosage was more than 2 wt%, which was most probably due to the limited solubility of the Ca(OH)_2_ in PHL. The removal of lignin increased by about 50% when Ca(OH)_2_ dosage was increased to 2%. It was reported that calcium could generate complexes with lignin, which implied that the complexation between lignin of PHL and dissolved calcium ions might have contributed to the removal of lignin [15]. In addition, the adsorption of lignin onto the Ca(OH)_2_ particles might have contributed to the lignin removal from PHL [3, 15].

The removal of furfural increased by 49% at 1.5 wt% of Ca(OH)_2_ in PHL. Furfural in the PHL was all removed with further increasing Ca(OH)_2_ dosage. The reason might be that furfural was oxidized under alkaline conditions. On the other hand, with increasing Ca(OH)_2_ dosage from 0 to 2 wt%, the acetic acid concentration increased from 6.2 to 12.9 g/L, which was due to the cleavage of acetyl groups of hemicellulose dissolved in PHL [15]. A further increase in Ca(OH)_2_ dosage decreased the acetic acid concentration slightly. This change was presumably due to the adsorption of acetic acid onto calcium hydroxide particles as reported earlier [3, 15].

Interestingly, the increase in Ca(OH)_2_ dosage to more than 2 wt% did not improve the removal of organics from PHL, which implied that the pH change of PHL and complexation between the PHL constituents and the dissolved Ca(OH)_2_ played more important roles than physical adsorption of organic materials on undissolved calcium oxide.

Ca(OH)_2_ treatment had a negligible effect on xylosugar removal when the Ca(OH)_2_ dosage was below 1.5 wt% (Fig. 2b). However, with further increase of Ca(OH)_2_ dosage from 1.5 to 4 wt%, the removal of xylosugar was significantly increased from 1 to 58%. One of reasons for this phenomenon was attributed to the hydroxide-catalyzed degradation reactions of xylosugars [25], and another reason might be the adsorption of xylosugars onto the calcium hydroxide particles, especially at a high Ca(OH)_2_ dosage [15]. Therefore, for the purpose of removing lignin with minimum loss of xylosugars, the optimal dosage of 1.5 wt% Ca(OH)_2_ was selected for further analysis.

**Fig. 3:**
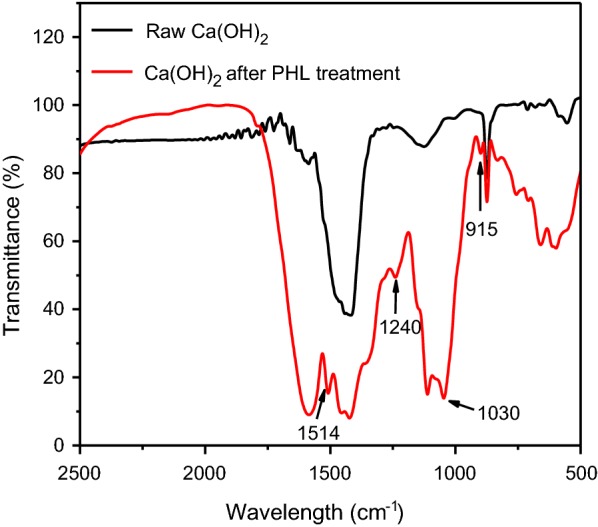
FTIR spectrum of the treated and untreated Ca(OH)_2_

The functional groups of the treated and untreated Ca(OH)_2_ were analyzed by the FTIR (Fig. 3). Several peaks appeared on the spectrum of Ca(OH)_2_. Peaks at 1514 and 915 cm^−1^ could be ascribed to the vibration of the aromatic skeletal and the C–H bending of aromatic nucleus, respectively [26]. The peaks at 1460 cm^−1^ could be attributed to the deformation of C–H (methyl and methylene) [27]. In addition, the peaks at 1240 and 1030 cm^−1^ could be assigned to the C–H and C–O bending of guaiacyl ring [28]. It was confirmed from FTIR spectrum that a portion of lignin and lignin-derivatives were adsorbed on Ca(OH)_2_ particles in the process of Ca(OH)_2_ treatment.

### 1st AC adsorption

**Fig. 4:**
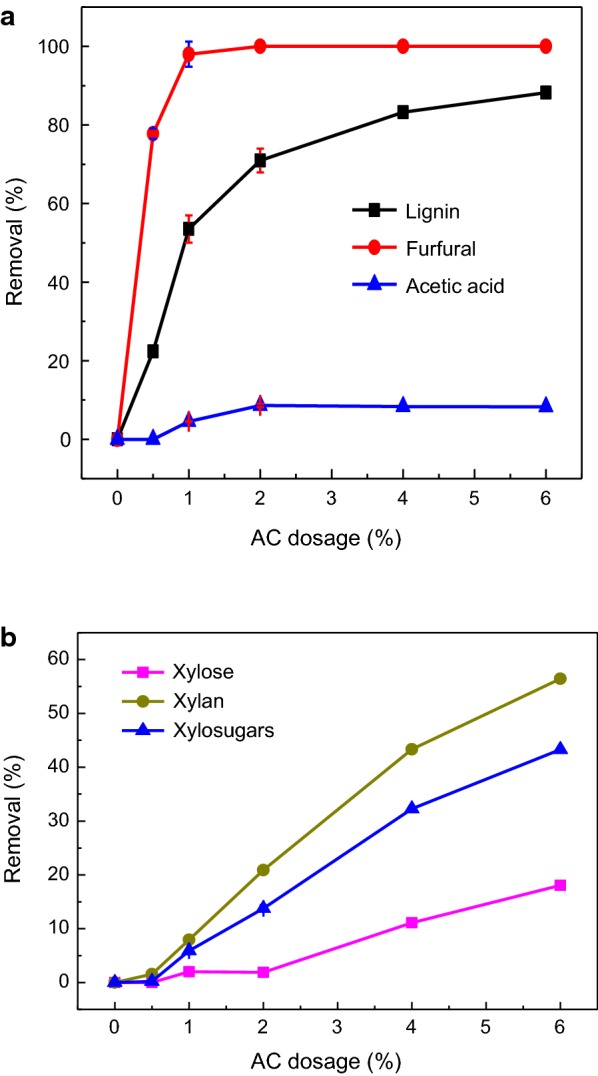
Effect of AC dosage on the constituents of PHL-1st (**a** removal of lignin, furfural and acetic acid; **b** removal of xylose, xylan and xylosugars)

The PHL-1st (i.e., PHL of the Ca(OH)_2_ treatment) was treated with AC for removing contaminants from PHL and the results are shown in Fig. 4. It can be seen from Fig. 4a that the removal of lignin and furfural were 53.5% and 98.0%, respectively, at 1 wt% AC dosage. Further increase in the AC dosage had a slight effect on the contaminant removal. However, AC treatment had no effect on the removal of acetic acid.

Figure 4b showed that the removal of xylosugars was significantly increased with the AC dosage increase, which is due to its higher adsorption on the AC surface. At a lower AC dosage (< 1.0 wt%), there are only limited adsorption for lignin, furfural and xylosugars on activated carbon. Lignin and furfural are more hydrophobic than xylosugars, and thus they were easy to be removed by adsorption [4, 16]. Almost all of lignin and furfural were removed at a higher dosage of AC at the expense of some xylosugar removals, which implied that the selectivity of AC adsorption decreased.

The removal of xylosugars was mainly attributed to their adsorption onto AC. As reported earlier, oligosugars have lower solubility and higher tendency for isolation from solutions [1]. It was well illustrated that xylan has higher value than xylose for pharmaceutical and food applications [29]. Hence the optimal AC dosage was selected as 1 wt% based on the minimum of xylan removal.

**Fig. 5:**
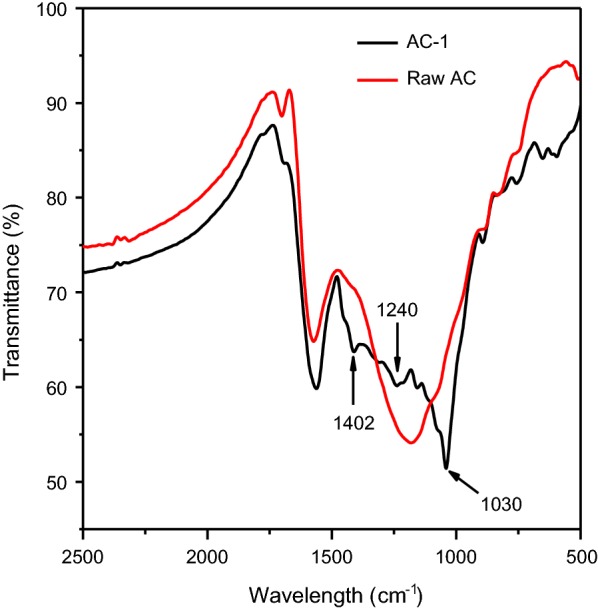
FTIR spectrum of the untreated AC and treated AC-1

The treated AC-1 and untreated AC were analyzed by FTIR, and the results are shown in Fig. 5. It can be seen that several peaks appeared after adsorbing organics on AC-1. The peak at 1420 cm^−1^ is ascribed to the carboxyl vibration of sodium carboxylate. The peaks at 1240 and 1030 cm^−1^ could be assigned to the C–H and C–O bending of guaiacyl ring [28]. FTIR spectrum illustrated that parts of lignin and lignin-derivatives were adsorbed onto AC in the AC adsorption process.

### Laccase assisted AC treatment

**Fig. 6:**
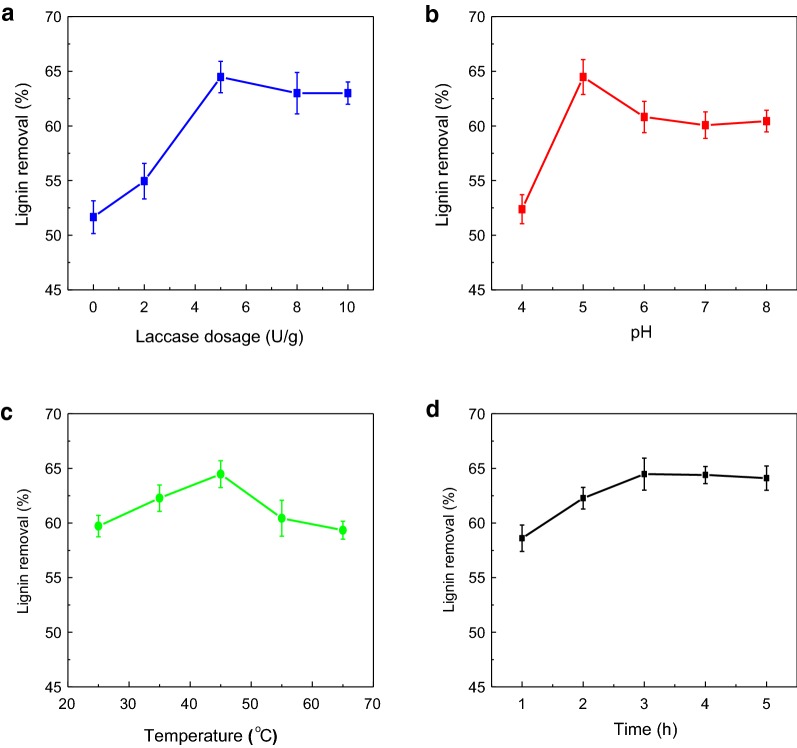
Effect of laccase treatment on lignin removal of PHL-2nd. The other treating conditions: **a** pH 5, temperature 45 °C and time 3 h; **b** laccase dosage 5 U/g, temperature 45 °C and time 3 h; **c** laccase dosage 5 U/g, pH 5 and time 3 h; **d** laccase dosage 5 U/g, pH 5 and temperature 45 °C

The aim of laccase assisted AC treatment is to polymerize the low molecular weight lignin of PHL-2nd by laccase so that their adsorption on AC is improved. The optimal laccase dosage, pH, treatment temperature and time were studied in the laccase treatment process, and the results are shown in Fig. 6. As can be seen, the maximum lignin removal was obtained at a 5 U/g of laccase dosage and pH 5, respectively (Fig. 6a). The laccase treatment was effective in a wide range of pH from 4.8 to 8 (Fig. 6b). The optimal temperature for laccase treatment was 45 °C and a higher temperature reduced the lignin removal rate. The optimal reaction time for laccase treatment was 3 h (Fig. 6d), and an increase in the treatment time did not affect the lignin removal. Lignin polymerization reaction is a radical–radical coupling reaction that would start by the laccase oxidation of phenolic end groups [30]. Thus, laccase facilitated the polymerization of lignin and the subsequent isolation of polymerized lignin due to its low solubility [1]. The optimal conditions of laccase treatment were 5.0 U/g of laccase dosage, pH 5, 45 °C and 3 h based on the maximum lignin removal achieved in this process.

The molecular weight of lignin of the PHL treated and untreated by laccase were analyzed as listed in Table 2. The molecular weight of lignin in PHL-2nd was too small to be determined, and thus is not reported. The results showed that the molecular weight (Mw) of lignin obtained under optimized conditions was 127% larger than that before laccase treatment, which supported the conclusion that lignin grafting/coupling reactions were induced by laccase [24]. It can also be seen in Table 2 that a high temperature (60 °C) made laccase deactivated, confirming the results shown in Fig. 6c. In addition, by increasing the treatment time from 3 to 24 h, the molecular weight of lignin increased slightly, the weight average molecular weight (Mw) increased from 6218 to 7418 g/mol, while its number average molecular weight (Mn) negligibly changed. The reason might be due to the fact that the molecular weight of lignin became sufficiently large in polymerization process that caused the steric hindrance for further extension of polymerization at a laccase treatment prolonged time [12].

**Table 2:** Molecular weight of lignin in PHL treated and untreated with laccase

Lignin	Mn (g/mol)	Mw (g/mol)	Mw/Mn
Before laccase treatment in PHL-2nd	3065	4909	1.60
Laccase treated under optimized conditions in PHL-3rd	3705	6218	1.67
Laccase treated for 24 h in PHL-3rd	3690	7418	2.01
Laccase treated at 60 °C in PHL-3rd	3339	5518	1.65

*Mn* number average molecular weight, *Mw* weight average molecular weight, *Mn/Mw* polydispersity

**Fig. 7:**
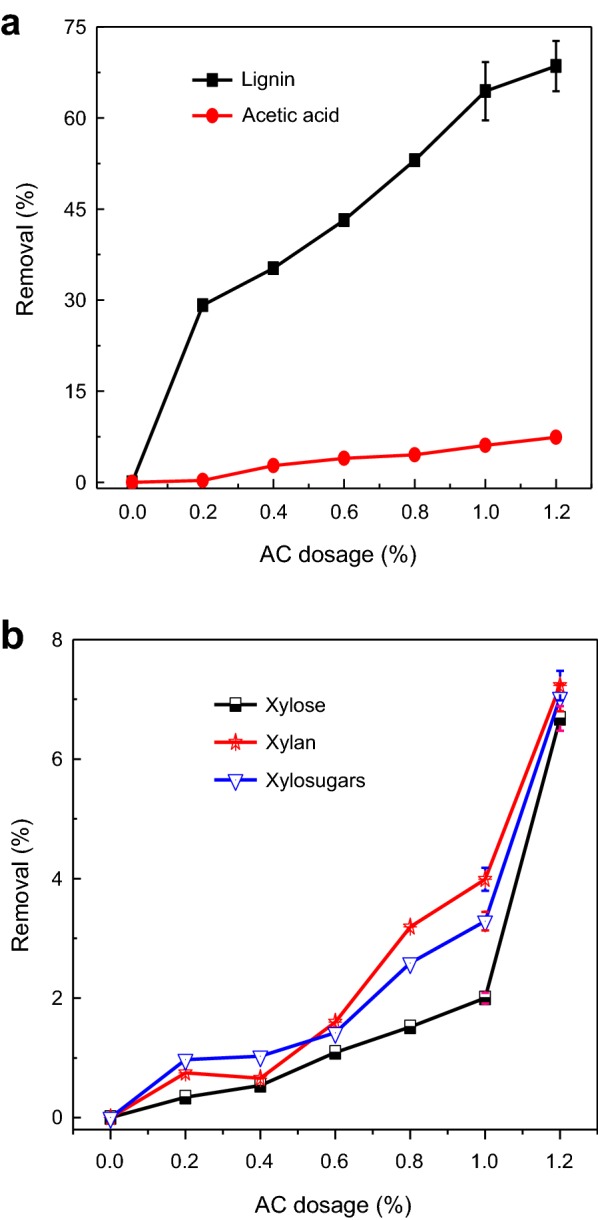
Effect of AC dosage on the removal of lignin and acetic acid (**a**) and xylosugars (**b**) from PHL-3rd

PHL-3rd was obtained under the optimal laccase treatment conditions. PHL-3rd was treated in the 2nd stage of AC adsorption at different AC dosages, and the results are shown in Fig. 7. It can be seen that the removal of lignin, acetic acid and xylosugars from the PHL-3rd increased by 64%, 6% and 3%, respectively, with increasing the AC dosage from 0 to 1 wt%. However, with further increase in the AC dosage, the removal of lignin and acetic acid slightly decreased, while the xylosugars removal increased rapidly. Therefore, the optimal AC dosage was of 1.0 wt%, resulting the maximum lignin removal and the minimum xylosugars removal from PHL.

The changes in the specific surface area and pore volume of the treated AC and the raw AC are listed in Table 3. The total specific surface area and pore volume of AC-2 and AC-3 decreased significantly compared to those of raw AC as lignin adsorbed on their surfaces and decreased their micropore surface and volume. However, the mesopore volume and its surface area changed slightly compared to micropore.

**Table 3:** Specific surface area and pore volume of ACs

AC	Surface area (m^2^/g)			Pore volume (cm^3^/g)		
	*S*_BET_	*S*_micr_	*S*_meso_	*V*_BET_	*V*_micr_	*V*_meso_
Raw AC	1185.4	707.7	435.5	1.1	0.3	0.7
AC-2	173.0	0	172.3	0.3	0	0.3
AC-3	107.4	0	105.7	0.2	0	0.2

*AC-2* precipitates obtained by treating PHL-2nd, *AC-3* precipitates obtained by treating PHL-3rd

The hydrodynamic diameters of components in PHL-3rd are listed in Table 4. It can be seen that the constituents of the PHL-3rd were in the size of 200–500 nm. As a result, the constituents of the treated PHL influenced the pore characteristics of micropores of AC and its adsorbing efficiency. Therefore, their adsorption affected the pore characteristics of micropores, however, the amount of adsorbed components and the size of these components were such that they did not fully occupy all the space and area in the mesopores [1, 9]. In addition, compared with AC-2, the mesopore surface area and volume of AC-3 were reduced more dramatically. Therefore, laccase treatment made larger lignin polymers that they adsorbed on AC more effectively.

**Table 4:** Hydrodynamic size of the components from different PHLs

Samples of PHL	Particle size (nm)
Origin PHL	342–825
PHL-1st	255–458
PHL-2nd	255–342
PHL-3rd	255–531
PHL-4th	< 21

The hydrodynamic sizes of the components in the PHL obtained from different treating steps are shown in Table 4. The results showed that the particle size of PHL reduced after treating by Ca(OH)_2_ and 1st AC adsorption stage. However, laccase treatment increased the particle size due to laccase-induced polymerization of lignin. The subsequent 2nd AC adsorption stage treatment led to the removal of almost all lignin. Therefore, the particle size of components of PHL-4th was very small.

### Overall performance

Table 5 shows the concentration of lignin, acetic acid, furfural and xylosugars at each treatment step. It can be seen that, after Ca(OH)_2_ treatment, the concentration of lignin and furfural of PHL decreased by 41.2% and 47.4%, respectively. The concentration of xylose and xylan changed negligibly and the concentration of acetic acid increased twofolds, which showed the high selectivity of Ca(OH)_2_ treatment process. The 1st AC treatment and laccase assisted 2nd AC treatment were effective in removing lignin and furfural, but inefficient in removing acetic acid. Overall, the concentration of lignin decreased by 90.7% and furfural was entirely removed from PHL. Compared to the original PHL, the concentration of xylosugars decreased by 9.9% (12% xylan and 5.7% xylose). In another work, the application of polydimethyl diallyl ammonium chloride (PDADMAC) led to 36.8% sugar removal and 70.3% lignin removal [14]. The PHL treatment of lime-resin resulted in 95% lignin removal and 21.2% sugar removal [31]. Comparatively, the combination of Ca(OH)_2_, AC and laccase treatment process was more effective in this work.

**Table 5:** Effect of different treating steps on the concentration of PHL components

PHL	Lignin (g/L)	Acetic acid (g/L)	HMF (g/L)	Xylose (g/L)	Xylan (g/L)	Xylosugars (g/L)
Origin PHL	9.7 ± 0.49	6.2 ± 0.26	1.9 ± 0.1	12.3 ± 0.68	23.3 ± 0.79	35.6 ± 1.47
PHL-1st	5.7 ± 0.29	11.7 ± 0.59	1.0 ± 0.04	12.1 ± 0.56	23.1 ± 0.99	35.2 ± 1.55
PHL-2nd	2.6 ± 0.17	11.2 ± 0.34	0.1	11.8 ± 0.47	21.3 ± 0.89	33.1 ± 1.36
PHL-4th	0.9 ± 0.07	12.7 ± 0.55	0	11.6 ± 0.52	20.5 ± 0.98	32.1 ± 1.5
Removal (%)	90.7	–	100	5.7	12.0	9.9

### Proposed process for lignin removal from PHL

**Fig. 8:**
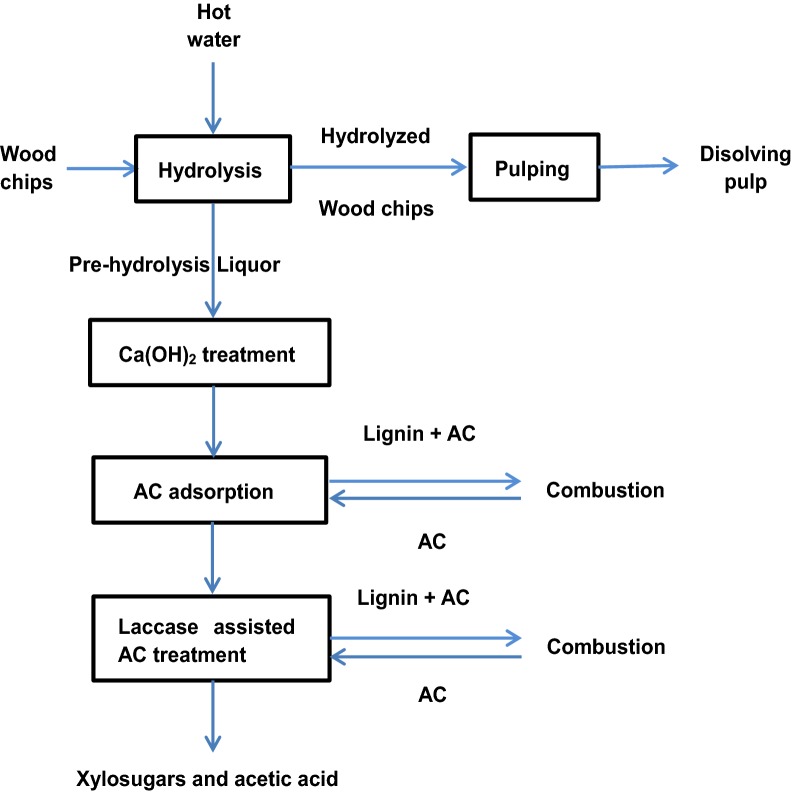
Proposed process for contaminant removal of PHL and xylosugars and acetic acid production

Figure 8 shows a proposed process for contaminant removal from PHL and xylosugars production. This process includes Ca(OH)_2_ treatment, AC adsorption, laccase assisted AC treatment and xylosugar purification, which were proposed based on the results obtained in this work. There were two advantages for using Ca(OH)_2_ treatment in the first step: (1) pH adjustment of PHL and (2) contaminant removal. The 1st AC adsorption could further remove macromolecular lignin from the PHL that was already treated by Ca(OH)_2_ (i.e., from PHL-1st). Lignin would be effectively adsorbed on AC, and the treated AC could be activated and recovered for reuse in a reactor via oxidation using different chemicals. The combination of laccase and 2nd AC treatment could help further remove the residual lignin and furfural from the PHL. The AC treated in the 2nd AC stage can be combined with that treated in the 1st AC stage to be activated and recovered for reusing. The laccase and lignin can be desorbed from AC, e.g., via steam or alkaline treatment [32, 33]. The purified PHL with xylosugars and acetic acid could be used to produce value-added products, such as ethanol and xylitol [34, 35]. The presence of acetic acid in PHL would help the production of xylose [36, 37].

Despite promising results, the order of process steps may be altered for better outcomes and the authors are currently working on this aspect. After optimizing the process, economic analysis should be conducted to validate the feasibility of the developed process.

Analysis of elements and property of AC from the different treating steps are shown in Table 6. It can be seen that the high heating value (HHV) of the treated AC increased due to the extra thermal energy from burning lignin and other components adsorbed onto AC. The adsorption of lignocelluloses increased the overall mass of the AC-based product and the elemental compositions of the treated AC changed compared to those of the untreated AC. In addition, the adsorbed lignocelluloses may have different structures, which may be another reason for different HHV of the treated AC [3].

**Table 6:** Analysis of elements and property of AC from the different treating steps

Samples of AC	Ash (%)	C (%)	H (%)	O (%)	N (%)	S (%)	HHV (MJ/kg)	Total HHV for 1 kg of AC (MJ)
Raw AC	3.4	77.7	2.9	16.0	0.3	0.2	28.8	28.8
AC-1	5.4	65.1	3.5	25.7	0.3	0.1	24.1	37.7
AC-3	6.3	64.4	3.3	25.7	0.3	0.1	23.6	31.6

Although the results showed promising contaminant removals and a strategy for energy recovery, the impact of the process on down steam fermentation processes for conversion of xylosugars to value-added products has not been examined in this work. Also, economic analysis and feasibility studies need to be conducted to determine the overall profitability of the proposed process.

## Conclusions

A novel process for separating contaminants from PHL was attested experimentally at a lab scale. Ca(OH)_2_ treatment was effective in selectively removing lignin and furfural from PHL without influencing the concentration of xylosugars. The 1st AC adsorption could remove lignin and furfural effectively and was ineffective in removing acetic acid. Xylan was more adsorbed on AC than xylose. Interestingly, the in situ system of laccase and AC improved the removal of contaminants by 17.6%, and this improvement was originated from the polymerization tendency of laccase for lignin. Overall, the removal rates of lignin, furfural and xylosugars were 90.4%, 100% and 9.9%, respectively. Based on the results, a new process was proposed for purifying PHL for downstream sugar-based biorefineries and generating heat that can be potentially used in the proposed process.
